# Survey of aflatoxins in Kashkineh: A traditional Iranian food

**Published:** 2011-09

**Authors:** M Mardani, S Rezapour, P Rezapour

**Affiliations:** 1 Department of Nutrition, Lorestan University of Medical Sciences; 2 Genetics, Lorestan University of Medical Sciences; 3 Lorestan University

**Keywords:** Aflatoxins, Kashkineh, HPLC, Khorramabad

## Abstract

**Background and Objectives:**

Aflatoxins are mycotoxins produced by *Aspergillus flavus* and *Aspergillus parasiticus* that can contaminate human and animal foods, including corn, wheat, rice, peanuts, and many other crops resulting in the illness or death of human and animal consumers. The aim of this study was to detect aflatoxin B1, B2, G1, G2 and total aflatoxin in Kashkineh, a traditional Iranian food.

**Materials and Methods:**

This survey was conducted to detect aflatoxins on 41 samples of Kashkineh. The samples were randomly collected from traditional bazaars and supermarkets of Khorramabad city of Iran. The presence and quantity of aflatoxins was determined by high performance liquid chromatography (HPLC).

**Results:**

The average concentrations of AFB1, AFB2, AFG1, and AFG2 in all samples and in a mixed sample of all samples were not detectable (ND). The only sample that showed aflatoxin contamination was sample number 29 of which the AFB1 concentration was 0.64 ng/g.

**Conclusion:**

Although some people believe Kashkineh is carcinogenic due to toxins, this study showed kashkineh is not contaminated with aflatoxins.

## INTRODUCTION

Aflatoxins are mycotoxins produced by *Aspergillus flavus* and *Aspergillus parasiticus* that can contaminate human and animal foods (1). Aflatoxins refer to a group of four mycotoxins (B1, B2, G1 and G2) produced primarily by two closely related fungi, *Aspergillus flavus* and *Aspergillus parasiticus*. Many studies indicate four types of aflatoxins, (B1, B2, G1 and G2) and their two metabolites M1 and M2 can contaminate foods, including corn, wheat, rice, peanuts, and many other crops resulting in the illness or death of human and animal consumers (2). The strains of *A. flavus* produce two aflatoxins, B1 and B2, but most strains of *A. parasiticus* could produce all four toxins (3).

Aflatoxin B1 (AFB1) is the most commonly occurring toxic compound (4). AFB1 can increase the incidence of disease and reduce productive efficiency. Some of the gross effects of AFB1 can include: intake reduction or feed refusal, reduction in nutrient absorption and metabolism, digestive disorders including hemorrhage and necrosis. Toxicity occurs at the cellular level. AFB1 could cause many DNA changes, cell deregulation, cellular changes and death (5, 6).

Aflatoxin infection occurs in crops during plant growth, maturation, harvesting and processing of grains. Infection can be induced when maturing corn is under drought conditions and prolonged periods of hot weather. Contamination can also occur during storage of the crop if moisture and relative humidity, oxygen availability, damaged or broken grain kernels are allowed to exceed critical values (7).

Kashkineh is a kind of Iranian traditional food that is used as an anti-cold food in autumn and winter seasons by most people of Lorestan and other western provinces of Iran. It is called Tarkhineh in some cities of Iran such as Sanandaj, Kermanshah and Ilam. This traditional food is always made in summer from washed and grinded wheat that has been soaked and mixed with churned sour yogurt (named dough in Persian). In some cases with dried pennyroyal leaves and some turmeric and salt are added. Then after 5 to 7 days, Kashkineh is dried under the sun and will be sold in stores. During the preparation and storage of this food, some fungi may grow and ultimately secrete different types of Aflatoxin.

Since aflatoxins are potential carcinogens, their quantity in food and feed is closely monitored and regulated in most countries. The European Union has a maximum level of 2 µg/kg for aflatoxin B1 and 4 µg/kg for total aflatoxins in crops (8, 9). However, the Iranian standard institute has a maximum level of 5 ng/g for aflatoxin B1 and 15 ng/g for total aflatoxins in crops. Due to lack of research for the contamination of Kashkineh with aflatoxins, the purpose of this study was to investigate and detect the rate of aflatoxins B1, B2, G1 and G2 in Kashkineh that is sold in supermarkets and traditional bazaars of Khorramabad city of Iran.

## MATERIALS AND METHODS

41 samples of kashkineh were randomly collected from the supermarkets and traditional bazaars of Khoramabad city, Iran, from February 2008 to April 2008. The collected samples were stored in plastic bags at –20°C until they were analyzed.

HPLC analysis was performed to detect Aflatoxins (AFB1, AFB2, AFG1, and AFG2) and each Aflatoxin level such as AFB1, AFB2, AFG1, and AFG2 was determined. The size of each sample was 250 grams that two sub samples, 50 grams, of each sample were ground in a laboratory mill to pass a 1.0 mm, then, screened and mixed accurately to ensure homogeneity. Then, a sample test was extracted with 5 grams of sodium chloride and methanol-water (80 + 20); the extract was filtered by filter 0.45 µm (PTFE) , diluted with phosphate-buffered saline solution, filtered on a microfiber glass filter. Finally, 100 µl of the samples with 100 µl of each standard of AFB1, AFB2, AFG1 and AFG2 were injected in the HPLC.

Calibration curves were prepared and determined as described in the Official Journal of the European Communities (1992; N° L327/54) (14). After each test, the column was washed with deionized water to remove interfering compounds. The recoveries were done in duplicate. The average recovery was 98.0%.

## RESULTS

In this study, 41 Kashkineh samples were analyzed twice for the levels of AFB1, AFB2, AFG1, and AFG2 by HPLC. The average concentrations of AFB1, AFB2, AFG1, and AFG2, in all of the samples and in a mixed sample of all, were non detectable (ND). The only sample that showed aflatoxin contamination was sample number 29 of which, AFB1 concentration was 0.64 ng/g (Table 1). According to National Standard of Iran (No: 6872), permitted rate of AFB1 in wheat and its products is 5 ng/g and for total Aflatoxins (B1, B2, G1, G2) is 15ng/g. Having an AFB1 level under the minimum level, the sample number 29 was considered as negative. Due to AFB1 level of sample number 29 under minimum limit, it was considered as negative.

**Table 1:** AFB1, AFB2, AFG1, and AFG2 levels in kashkineh samples (n = 41) obtained by HPLC.

Number of sample	Aflatoxins (ng/g)				
	AFB1	AFB2	AFG1	AFG2	Total
1-28	ND	ND	ND	ND	ND
29	0.64	ND	ND	ND	ND
30-41	ND	ND	ND	ND	ND
Mixed of all	ND	ND	ND	ND	ND
Acceptable limit	5ng/g	-	-	-	15ng/g

## DISCUSSION

Contamination of wheat and other food products with aflatoxins is a public health concern because of the ability of aflatoxins to cause human and animal diseases (10). Kashkineh is made from broken wheat (groats) that are soaked in dough for 5-7 days and dried in sunshine, and is finally stored for use in cold seasons.

Our results showed that none of the 41 samples were contaminated with aflatoxin B1, B2, G1 and G2 except for sample number 29 that was contaminated with aflatoxin B1. However, its contamination was below the standard rate.

Many investigations have pointed that aflatoxin contamination was not found in many food products such as corn products, corn, peanuts, buckwheat flour, dried buckwheat noodles, rice, or sesame oil or rice (11, 12).

The results of Baydar and colleagues study pointed out that the contamination of the aflatoxin and ochratoxin in the retail ground samples in Ankara, Turkey, were under the permitted levels. Nevertheless, they suggested that because of the overall daily intake, the mycotoxins should be considered for evaluation of their health risk (13).

The study of breakfast cereals for aflatoxin contamination has showed only 4% of the breakfast cereals and 1% of the infant cereals had aflatoxin B1 levels exceeding 0.1 ng /g which is the European Union maximum limit for aflatoxin B1 in baby foods and processed cereal-based foods for infants and young children (14). However, Breaicu et al have reported that 58.14% of total samples of different cereals were contaminated The highest number of contaminated samples was found to be in the wheat samples (62.5%) that may be due to culture condition or possibly weather condition of a particularly wet summer (15).

AFB1 concentrations of 5 µg/kg or more were recorded in 40.3% of the samples, and concentrations above the Indian permissible regulatory limit of 30 µ/kg were found in 16% of the samples (16).

The study of aflatoxin contamination in corn, wheat and poultry feeds from Morocco by HPLC and IAC showed that the contamination of 10% of samples of corn was higher than the limit set by EU regulations for AFB1 and total AF (17). Tunisian foods study (18) have shown that 50.5% of foods such as cereals, cereal products, spices, dried fruits and sorghum are mainly contaminated with aflatoxin B1. The study of Cho and his colleague has shown that 12 samples of spices (red pepper, curry and ginger products) were contaminated with aflatoxins B1,B2,G1 and G2, although black pepper and cinnamon were detected below the limit of detection (19).

The lack of contamination of Kashkineh with aflatoxin could be due to the purification of wheat and its products, because of their dry condition after harvesting. According to Behfar study all wheat flour samples that were collected from the wheat factory of Ahvaz city in Iran did not exceed the maximum permissible level of total aflatoxins for food (20). It seems that the traditional techniques for preparing and conserving Kashkineh are the optimal methods for doing so; namely exposure to temperatures over 40°C with very low humidity for drying and direct subjection to the sun, could prevent mould growth and aflatoxin production.

As Bennett and Clich in their review about mycotoxins have pointed one of the methods for controlling mycotoxins is good agricultural practice and sufficient drying of crops after harvesting (21). The incidence of AFs in dried fruits and nuts could be avoided or at least decreased if good agricultural and manufacturing practices from harvesting to processing were used (22). Moreover, using washed wheat to make Kashkineh caused reduction of aflatoxin in it. Also, Mutungi et al have reported de-hulling the grains significantly decreased (P < 0.001) aflatoxin levels to between 6.8 ng/g and 182 ng/g (10).

Some studies have reported that, aromatic organic compounds of spices can control and inhibit the fungal growth and production of aflatoxins. (23–27). Also they have pointed that, their antimicrobial properties have been found to be mostly due to the presence of alkaloids, phenols, glycosides, steroids, essential oils, coumarins and tannins (23, 28, 29).

Ficker *et al*., found that the extract of *Curcuma zedoaria* have pronounced inhibitory activity against a wide variety of human pathogenic fungi (30). Therefore addition of dried pennyroyal leaves to Kashkineh precursors that have alkaloids, phenols, glycosides, steroids, essential oils, coumarins and tannins could be a reason for inhibition of fungal growth and contamination with aflatoxins.

Furthermore, the acidic environment of dough, fermented milk with lactic acid bacteria (LAB), yoghurt and dough (churned sour yogurt) in particular, are popular carriers of probiotics (31) and its fermentation may inhibit fungi growth and aflatoxin secretion in Kashkineh.

Addition of turmeric to Kashkineh may cause affect as one of the inhibitory factors of fungi growth or aflatoxin secretion. The antifungal properties of turmeric have been described and documented by many studies (26, 30, 32–34).

According to the results of this study, we can conclude that Kashkineh is not contaminated with aflatoxins. Due to it's preparation it seems that fungi can not growth in this food.
